# GPs' decisions on drug treatment for patients with high cholesterol values: A think-aloud study

**DOI:** 10.1186/1472-6947-4-23

**Published:** 2004-12-13

**Authors:** Lars Backlund, Ylva Skånér, Henry Montgomery, Johan Bring, Lars-Erik Strender

**Affiliations:** 1 Center for Family Medicine, Karolinska Institutet, Alfred Nobels allé 12, SE-141 83 Huddinge, Sweden; 2 Department of Psychology, University of Stockholm, Sweden; 3 Statisticon AB, Östra Ågatan 31, SE-753 22 Uppsala, Sweden

## Abstract

**Background:**

The purpose was to examine how General Practitioners (GPs) use clinical information and rules from guidelines in their decisions on drug treatment for high cholesterol values.

**Methods:**

Twenty GPs were presented with six case vignettes and were instructed to think aloud while successively more information about a case was presented, and finally to decide if a drug should be prescribed or not. The statements were coded for the clinical information to which they referred and for favouring or not favouring prescription.

**Results:**

The evaluation of clinical information was compatible with decision-making as a search for reasons or arguments. Lifestyle-related information like smoking and overweight seemed to be evaluated from different perspectives. A patient's smoking favoured treatment for some GPs and disfavoured treatment for others.

**Conclusions:**

The method promised to be useful for understanding why doctors differ in their decisions on the same patient descriptions and why rules from the guidelines are not followed strictly.

## Background

The medical decision examined in our study is whether or not to initiate drug treatment for high cholesterol values. The topic has been the focus of much debate on the grounds that the proportion of individuals with elevated cholesterol values is high in most Western populations, and that the costs for treating all these people with drugs life-long would be enormous, with a marginal benefit in risk reduction for the majority of them [1-3]. The current Swedish guidelines [4] from 2003 are national applications of the recommendations on coronary prevention of the Third Joint European Task Force [5]. In sum, the national guidelines define a total cholesterol value above 5 mmol/l and/or an LDL value above 3 mmol/l as hypercholesterolaemia and the same values as the goals for treatment. As a comparison, the American guidelines are more aggressive in terms of treatment goals for patients with established coronary heart disease and they are more focused on the LDL levels [6]. The Swedish guidelines identify two group of patients that in general should be offered pharmacological treatment after a sufficiently long trial of life style intervention: the individuals with already established cardiovascular disease (so called secondary prevention cases) or diabetes. A third group that in general should receive medication are patients with familial hyperlipidaemia (FH). For the remaining individuals with a total cholesterol above 5 mmol/l and/or LDL above 3 mmol/l (primary prevention), the same guidelines suggest that the decision to recommend a drug or not should be based on an estimate of the combined risk stemming from the individual's different risk factors. More specifically, the recommendation is to make a numerical risk estimate of the risk for coronary heart disease (CHD) within the next ten years (or the risk projected to 60 years) with a cut-off value at 20%. Based on the results from epidemiological studies, algorithms for arriving at such risk estimates (e.g. Anderson et al. [7]) have been developed and are available, for instance, as charts in pocket format for doctors.

Thus, the decision-making task can be carried out as follows. The first step is to decide whether the patient case is an instance of secondary prevention, diabetes or FH, and if not, to estimate the numerical risk for coronary disease within ten years. A risk above 20% would justify pharmacological treatment, given that life style intervention has been tried for a sufficiently long period.

In this study we address the question of how General Practitioners (GPs), who manage most of the cholesterol testing and treatment in Sweden, make such decisions when guidelines are not physically available to them. We will try to highlight the decision-making by examining how it is affected by clinical variables describing the patient and by medical knowledge and decision rules on behalf of the doctors. The reason for studying decisions without access to written guidelines is that as experienced GPs (in the case of three of us), we have found that this is how decisions on cholesterol treatment are usually made. Furthermore, in a previous study concerning the ability of GPs to make numerical estimates of future cardiovascular risks, we asked the GPs if they had access to any tool for making numerical risk estimates at their clinic [8]. Only nine out of 84 respondents said they had such a tool. GPs' judgments regarding cholesterol treatment have been studied previously using Clinical Judgment Analysis, CJA (for a description of this research paradigm, see Cooksey [9], for an overview of the medical applications, see Wigton [10]). The variation among doctors with respect to which information about the patient influenced their decisions most (strategies) seemed to be high [11], and the strategies were not in accordance with the guidelines for a substantial proportion of the doctors [12]. The number of patient variables (cues) that influenced the decisions was two or three for most doctors [11,12]. About one-fourth of the doctors did not include coronary heart disease in the irjudgments [12], in spite of the central role of this risk factor according to the guidelines. The statistical modelling with CJA describes individual doctors' judgment strategies added together for a set of cases, but does not give direct information about the kind of rules or medical knowledge that the participants use in their decisions. In the present study we used the think-aloud technique [13] in order to learn more about the use of medical knowledge and rules. In addition, with this technique we are better able to study the decisions for individual patient cases. In a previous paper, based on the present data [14], we coded the think-aloud protocols for preferences concerning two decision alternatives, prescribing a cholesterol lowering drug or not doing so. The codings proved to be reliable. They also appeared to be valid in the sense that there was good agreement on how think-aloud data and rating data, both concerning preferences for prescribing and not prescribing, described the decision process over time for different simulated patient cases. In the present study we link such preference data from think-aloud protocols to different kinds of information describing the patient cases. We also investigate how the use of rules and guidelines can be inferred from the think-aloud protocols.

Our first set of research questions concerned how different kinds of information about the patient (e.g. age, sex, previous diseases and laboratory tests) relate to the decision to prescribe a drug or not to do so. First, we estimated the importance of the individual information categories by counting the total number of times they had, according to the coding of the verbal protocols, been valued in a positive or negative direction in relation to the decision at hand. Second, in order to get an idea about why different doctors reach different decisions when presented with identical case information, we made the following analyses. For each of the patient cases separately, the subgroup who decided to prescribe was compared with those with an opposite decision regarding how often they valued different information categories. Third, to further understand how the participants differed in their judgments, we examined which kinds of specified information about a patient (e.g. male sex) are the most likely to lead to disagreement, i.e. to be judged in a positive direction by some participants and in a negative direction by others.

Disagreement about the evaluation of data on a given variable may result from different cut-off values, e.g. for the cholesterol variable. A certain value can be considered high for one participant and thus speaking for drug treatment. The same cholesterol value may be considered almost normal by another participant and thereby speaking against drug treatment for the same patient. The age variable may also be associated with disagreement due to different cut-off values. A higher age is generally associated with a higher risk, but there is a lack of evidence for the potential benefit of treating the oldest age groups, and this may introduce different cut-off values for different doctors.

Disagreement may also be caused by what might be called different perspectives. If we take smoking as an example all doctors should recognize smoking as a factor associated with an increase in future cardiovascular risk, and should accordingly make statements with a positive directionality for drug treatment. On the other hand, some doctors in some situations may regard actions aimed at smoking cessation as more beneficial than cholesterol reduction, which may give smoking a negative directionality in relation to drug treatment. Overweight can be regarded in the same way, i.e. as an indicator of drug treatment or as indicating change of life style as preferable to drug treatment. Thus, there are two alternative treatment philosophies – drug treatment or life style change – which in turn may be associated with opposite evaluations of the same data in relation to drug treatment. To the extent that these philosophies in fact are associated with different evaluations, one may regard them as different perspectives where certain data (e.g. smoking) are seen from different angles, as risk indicators or as entities that could be changed through patient's own efforts (i.e. by changing life style) as a means to treat the his or her health problem. The latter perspective may also be associated with somewhat moralistic evaluations, e.g. that overweight or smoking is the patient's own choice or own "fault", which in turn would decrease the inclination to initiate drug treatment. Some evidence for this conjecture comes from a CJA study by Evans et al [11]. They interviewed the doctors after the case presentations regarding which factors they thought had influenced their judgment most. The GPs stated that they were generally less likely to treat people who were overweight. Most were also less likely to treat smokers, but some had the opposite policy. Those less likely to treat smokers were also less likely to treat obese patients. The traditional medical risk factors like diabetes and hypertension may also be associated with either the risk increase perspective or an alternative perspective, where other variables than the cholesterol level are in focus for treatment. Such an alternative perspective should be more likely with a poor control of the blood pressure or diabetes parameters. As this was hardly the case with our case vignettes, and as the moralistic perspective is more likely with life style factors, we expected disagreement to be more frequent with life style variables than with traditional medical factors.

Our second set of research questions concerned the use of rules, and the concept of risk as shown in the verbal protocols. Six patient cases were chosen that included two high-risk patients (secondary prevention or diabetes) for whom the guidelines can be transferred to a simple decision rule (e.g. "patients with elevated cholesterol values and previous coronary heart disease should have drug treatment recommended"). Our question was how frequently such decision rules were in the verbal protocols and their content in relation to practice guidelines for elevated blood lipids. For the remaining four cases (primary prevention) no such simple guideline-based rule can be applied and instead, a numerical risk calculation is suggested. We examined the extent to which references to risk estimates were made in the think-aloud protocols. For both secondary and primary prevention cases we were interested in determining how the decisions corresponded to what is indicated by guidelines and risk algorithms.

In sum, our research questions concerned: Importance of information (which categories of information about the patients seem to be most important for the decisions?). Patterns of importance for "Yes" and "No" decisions (when each case is analyzed separately, can we get an idea of the reasons behind different decisions by comparing the information evaluation for doctors who chose prescription with those with the opposite decision?). Disagreement (which categories of information give rise to disagreement?). Use of rules (their frequency and contents). Risk estimation (for cases that should be decided by use of a numerical risk calculation, according to the guidelines, how frequent is a referral to the concept of risk estimation in the verbal protocols?).

Our approach to analyse think-aloud protocols in a medical decision task for the relative importance of different information categories and the amount of disagreement in the evaluation of these categories has not been tried before as far as we know. We believe that the results can be useful for understanding why doctors reach different decisions in response to the same patient cases and why they often do not act in accordance to guidelines. This knowledge should be useful as an aid to design guidelines and teaching.

## Methods

### Design

The 20 participants received the same six patient cases and the order of the cases was the same for all participants. Cases with "Yes"- and "No" decisions as the recommended treatment according to the guidelines were mixed as evenly as possible. Ten of the participants were randomly assigned to a condition where, in addition to thinking aloud, they also rated their willingness to prescribe a drug at regular intervals during each case. As was described in a previous paper [14], this group did not differ from the group without the rating task as regards the prescription decision or the directionality in the think-aloud data. In the present paper the think-aloud data from these two groups have been compiled, whereas the analyses of the rating data are confined to this previous paper.

### Participants

Twenty GPs working in the southern Stockholm area participated. There were 10 males and 10 females. Their ages varied between 34 and 60 years (M = 48.3) and they had practiced between one and 22 years (M = 11.4) as specialists in family medicine. A total of 36 doctors were contacted by telephone. They were selected so as to have a relatively even distribution across different districts in the area and according to gender, but the selection was not random. Twenty-four agreed to participate, but before the session four of these later declined to participate.

### Cases

Six clinical cases were selected from an original set of 40 authentic cases with cholesterol values above normal (at least 5.5 mmol/L). The original set was used in a Clinical Judgment Analysis design with a different sample of doctors and is described in Backlund et al [12]. Two of the cases, GM (with diabetes mellitus) and AR (with angina pectoris), were obvious high-risk patients and it would be reasonable to use the guidelines in a straightforward manner and recommend treatment for these cases (under the assumption that lifestyle modification had already been tried). Case SH was distinguished by the absence of risk factors other than a moderate increase in cholesterol level, and it would therefore be reasonable to refrain from drug treatment. In the remaining three cases (IS, TW and PU) additional risk factors existed such as smoking and hypertension, and the recommended line of management would be to let the decision be guided by a numerical estimate of the future risk for coronary heart disease. The presently available risk-charts that are referred to in the Swedish [4] and European guidelines [5] indicate a 10–20% ten-year risk for case IS (treatment not justified), and a 20–40% risk for case TW (treatment justified). For case PU the calculated risk is 10–20%, which would suggest refraining from drug treatment. However, case PU had a strong family history of coronary heart disease, which is an important risk factor although it is not directly included in the chart. A decision to prescribe a drug is probably justified for this case. Case SH had a calculated risk of 5–10%. For all six cases lifestyle intervention as well as advice concerning diet and exercise had been tried for at least six months before the visit in question.

The different kinds of clinical information presented on the six successive screens were divided time-wise in the same way for all six cases. The order in which this information was presented was arranged so as to be as realistic as possible in relation to clinical practice (including how patient cases are described in written referrals to other clinics and in clinical conferences and tutoring). Table 1 shows case IS as it was presented to participants in the study. All previously shown information about a case was repeated on the later screens to reduce and control for memory effects. This part of the text was placed at the top of the screen and was a different colour.

### Procedure

The study was conducted at the doctor's office or in a room nearby. All visits and recordings were made by one of the authors (LB). The cases were presented on a computer screen (Software Question Asker™ (QA) [15]. In the course of six screens, more clinical information was gradually added to the case. The participants were instructed that authentic cases of hypercholesterolaemia would be presented and that their task was to voice aloud all their thoughts about the case, and that each case would end with the question as to whether or not they would prescribe a drug for this patient. They controlled the shift to a new screen by using a mouse click. When the participant had finished a screen, he or she clicked on a "continue" button. If a participant was silent for more than 10–15 sec, he or she was reminded to voice aloud all thoughts about the information presented.

The study was approved by the local ethics committee.

### Response measures and coding of data

#### Decision

Each case ended with a screen with the following question: "Would you prescribe a cholesterol-lowering drug for this patient?" The participant responded by clicking on one of two response alternatives, "Yes" or "No".

#### Think-aloud protocols

The sessions were tape-recorded. A secretary then transcribed the recorded sessions into a written, word-by-word format. The protocols were segmented into statements. The next step was to categorize the statements into one of ten categories concerning the general characteristic of the statement (cp. Cognition Categories): Attention, Evaluation, Rule, Explanation, Action pharmacological treatment, Action non-pharmacological treatment, Action other, Want of information, Rating (valid for the ten participants with an additional rating task) and Other. The set of categories is described in more detail in Backlund et al [14]. Each statement was also assigned one of the values +, -, 0 or x, denoting directionality in relation to the decision task (to prescribe or not to prescribe). The majority of statements that could be assigned a positive or negative directionality had been categorized into either "evaluation" or "application of a rule". However, the most frequent outcome of the categorization was "attention" (the participant read the information aloud, or retrieved it from memory, with neutral or no reformulation), and no directionality could be assigned (i.e. coded as "x"). Zero directionality indicated an explicit statement that the information was neutral in relation to the decision. Finally, each statement was coded with respect to the information referred to. A certain information category could be coded more than once for a given doctor and a given case, unless we regarded the statement as a mere repetition (close in time and identically or almost identically phrased as an earlier statement). The original set contained 21 information categories within the areas of background data, medical conditions, previous diseases, lifestyle factors, physical examination and laboratory tests.

## Results

### Reliability of the coding system

Two of the authors (LB and YS) independently coded the protocols from the first six participants. Reliability was computed separately for directionality (+, -, 0 or x) and for information category (one of 21) as the percentage of statements that were coded into the same directionality/information category. For these first six participants, the inter-judge reliability was 92% for directionality and 94% for information category. The reliability measures were considered to be satisfactory and therefore only one of the authors (LB) performed the remaining coding.

### Information categories

The original set of 21 different information categories was reduced to eleven. When we ranked the information categories with regard to the frequency of positive or negative evaluations there was a great leap between weight (frequency 14 and rank eleven) and triglycerides (frequency four and rank twelve). We therefore excluded triglycerides and information categories with fewer evaluations. Examples of such excluded categories were information about physical examination of the heart and lungs (normal outcome for each of the cases) and test results concerning liver or thyroid function. The remaining eleven information categories were Cholesterol, LDL (low density lipo-protein), HDL (high density lipo-protein), weight, smoking, CHD (coronary heart disease), diabetes, hypertension, heredity, sex and age.

### Treatment decisions

**Figure 1:**
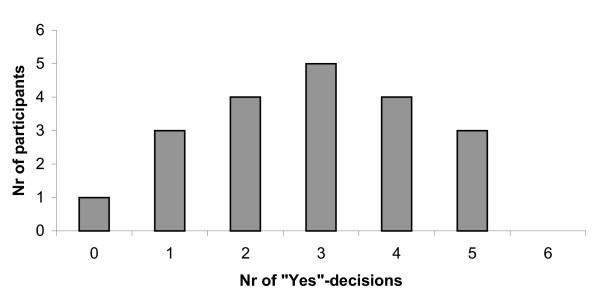
**The number of participants with 0–6 "Yes"-decisions **The number of participants with 0–6 "Yes"-responses, out of 6 possible, to the question whether or not to recommend drug treatment for the different patient cases.

Table 2 summarizes the information for each case as regards these eleven categories and shows the number of doctors who decided to prescribe a drug as well as the recommended decisions according to the Swedish guidelines. The frequency varied from zero (case SH) to 17 (case AR). "Yes"-decisions and "No"-decisions were equal in frequency when summarized over participants and cases. Figure 1 shows that the majority of doctors chose to prescribe for two, three or four of the six cases. No participant decided to prescribe for all six cases and one participant chose not to prescribe for any of the cases.

### Importance of information

**Figure 2:**
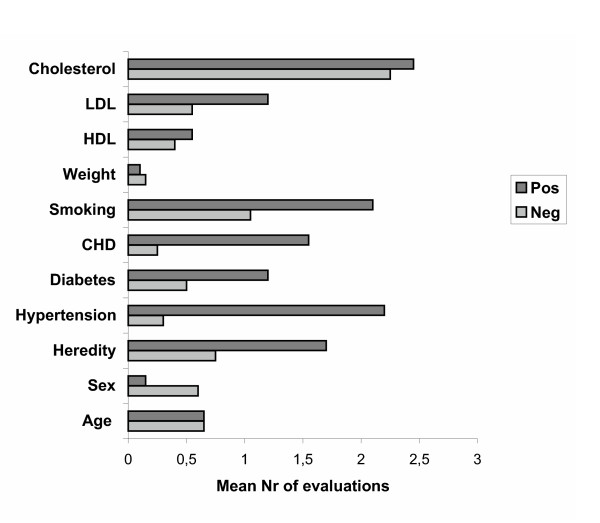
**Relative importance of the different information categories. **For each of the information categories, its importance for the decision to recommend drug treatment or not, was defined as the mean number of statements per participant that were coded with a positive or negative directionality in relation to the treatment decision.

Figure 2 shows that information about cholesterol was evaluated most frequently, both in the positive and the negative direction. It can also be seen that positive evaluations were more frequent than negative ones, except for sex, age and weight, which in part can be due how the final question was worded. For each of the 20 participants we calculated the number of evaluative statements (i.e. with a positive or negative directionality) for each of the eleven information categories as an index of the importance of the information category. A 2 (Direction: Positive/Negative) × 11 (Information category 1–11) × 6 (Case 1–6) ANOVA with three within-group variables and the number of evaluated statements as dependent variable was performed. The main effect of Information category was highly significant, F (4.4; 84.1) = 8.80, p < .01, as was Information category × Case interaction, F (9.1; 172.2) = 11.80, p < .01. (In ANOVA with repeated measures in this study, the degrees of freedom were adjusted according to the Greenhouse-Geisser Epsilon). Thus, as could be expected, the different information categories were evaluated unequally often, and the pattern of relative importance differed in the six individual patient cases. All other main effects and interaction effects were also significant with p < .01.

### Patterns of importance for "Yes" and "No" decisions

In the following, the six patient cases will be analysed separately. This may allow us to detect possible differences in the pattern of importance between different information categories for participants who decided to prescribe a drug and those who made the opposite decision. For each case the number of evaluative statements was the dependent variable in a 2 (Decision: Yes/No) × 11 (Information category 1–11) × 2 (Direction: positive/negative) ANOVA, with the first as a between-group variable and the latter two as within-group variables. The statistical effects are summarized in Table 3.

The main effect of Decision was not significant in any of the cases, indicating that there was no evidence of an association between the number of evaluative statements and decision outcome. For four of the cases the effect of Direction was significant, indicating that positive and negative statements were of unequal frequency. Except for case SH (the case for which all 20 participants decided not to prescribe), positive statements were more frequent than negative ones. For all six cases the different information categories were evaluated unequally often (i.e. main effect of Information). A significant Decision × Direction Interaction, indicating that the decision to prescribe or not to prescribe was associated with different distributions between positive and negative statements, was found in only two of the cases. Direction × Information Interaction was significant or nearly significant for all five cases, suggesting that the distribution of positive and negative directionality was unequal across the different information categories.

The most interesting part of these analyses, however, is whether different decisions were associated with different evaluative patterns across the information categories. Statistically, this corresponds to two- or three-way interaction effects including Decision and Information.

A significant Decision × Information interaction for a case would indicate that participants with a "Yes"-decision had their number of evaluative statements differently distributed across Information categories compared to participants with a "No"-decision, regardless of whether the direction was positive or negative. The three-way interaction includes the directionality of the statements as well. As can be seen from Table 3, most of these interaction effects were non-significant. However, the number of evaluations per patient case is probably too small to give enough power for such interaction effects. Three of the cases will be selected to illustrate how this approach may provide hypotheses about information strategies.

**Figure 3:**
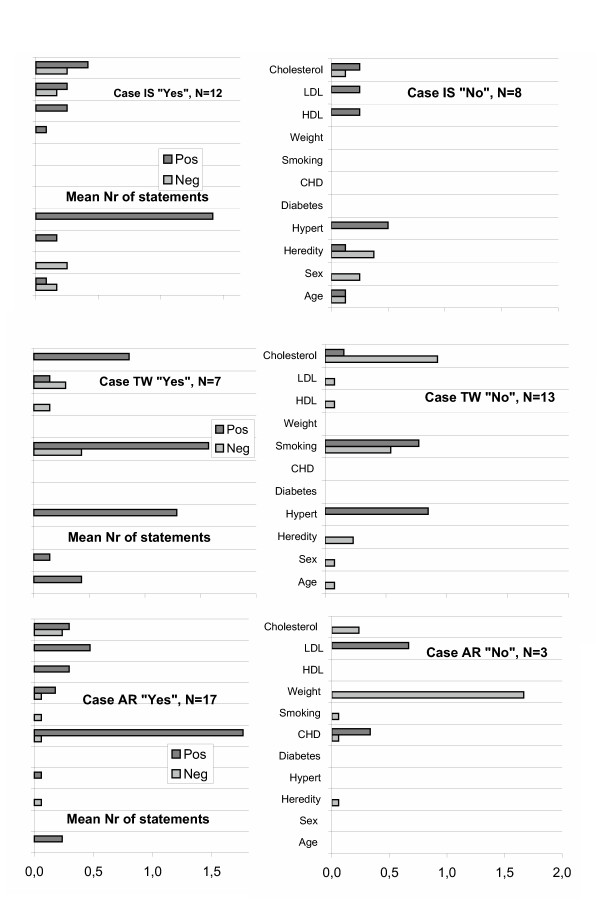
**Patterns of importance for "Yes" and "No" decisions. **As in Figure 2, the mean numbers of statements with a positive and negative directionality are shown for the 11 different information categories. In Figure 3, the patterns of positive and negative evaluations across the information categories are shown separately for the subgroups with "Yes" decisions and "No" decisions, respectively. Three of the six patient cases are shown.

Case IS represents a 67-year-old female with hypertension as a central risk factor in addition to her cholesterol elevation. She also had a modest heredity. As Figure 3 shows, the 12 participants who decided to prescribe had more positive evaluations of the central risk factor hypertension. In addition, the group who decided not to prescribe seemed to make negative evaluations of information about heredity, suggesting that this information may have been an "argument" against pharmacological treatment for some of the participants in the "No"-subgroup.

Case TW (Figure 3) represents a case with several risk factors in addition to cholesterol elevation (e.g. smoking and hypertension). An important difference between the groups may be that the "No"-group evaluated the patient's relatively low cholesterol level more often and in the negative direction in relation to pharmacological treatment.

Case AR (Figure 3) represents a case with CHD (in this case angina pectoris). A comparison between the response groups suggests that greater emphasis was put on CHD by the "Yes"-group, and at the same time there was a negative evaluation, as regards pharmacological treatment, of the patient's (over-) weight by some participants in the "No"-group.

Thus, an analysis of the response patterns for these three cases suggests that the "Yes" and "No"-groups differed not only in how much they evaluated the central risk factor(s) (in addition to cholesterol elevation) as favouring drug treatment but also in that the "No"-group seemed to have identified at least one information category as evidence against treatment. As regards the remaining cases, the evaluative pattern for case PU (young woman with severe heredity for CHD) could be interpreted in a similar way, with the patient's (young) age as an argument against drug treatment, whereas case GM (diabetic case) did not invite any such interpretation. For Case SH, no comparison between response groups could be made as all participants decided not to prescribe.

### Disagreement

We defined agreement as the degree to which the same information about a patient case was evaluated with the same directionality. We hypothesized that disagreement would be more common for information about lifestyle-related factors like smoking and weight than would be the case for medical conditions like hypertension and diabetes. There was only one case with a clear overweight and one case where the patient smoked, and the number of evaluative statements concerning these two information categories was therefore rather low. Regarding case TW's smoking, there were 21 statements with a positive direction and ten with a negative direction. For case AR's overweight there were three positive and six negative statements. For hypertension, each of the cases had either only positive or only negative directions (or no statements with directionality at all). The same pattern was found for diabetes and CHD, except that for diabetes two statements concerning case GM were negative compared to 24 statements with positive directionality, and for CHD (case AR) one statements was negative whereas 31 were positive. Thus, with minor exceptions the participants agreed on the evaluations of these three information categories. The data were consequently in line with our hypothesis.

There were few evaluative statements concerning the sex of the cases. With one single exception all statements were negative and concerned female cases, which is in line with the known lower risk for female patients to suffer from cardiovascular diseases. A corresponding tendency towards positive evaluations of the male cases was not clearly demonstrated in this material, which could suggest a possible bias in how sex is evaluated as a risk factor. As far as the age variable is concerned, there were both positive and negative statements (i. e. disagreement) for four of the six cases, which was in accord with our expectations. Among the information categories concerning laboratory values, cholesterol was evaluated most often by far, with a fairly even distribution of positive (total 46) and negative (total 39) statements. For at least four of the cases, there were approximately the same numbers of positive and negative evaluations of the same cholesterol value. In other words, according to our definition there is evidence of disagreement among participants in the evaluation of the different cholesterol values.

At the level of individual participants, there were eleven instances where doctors made both positive and a negative evaluation(s) of one case. Four of these eleven concerned smoking, two cholesterol, two LDL and one each of hypertension, coronary heart disease and diabetes.

### Use of rules

A total of 32 statements (i.e. not more than 1.6 per participant) were coded as rules. According to our judgment, 18 of these 32 statements were derived from or were compatible with the guidelines (including those rules that were not entirely correct in detail, e.g. regarding the cut-off limits for LDL and HDL) and twelve of these 18 were a more or less directly referring to secondary prevention or diabetes (e.g "He has angina pectoris and should be below 5 in cholesterol"). Examples of other contents for the statements coded as rules were age limit for cholesterol treatment, importance of looking for secondary hypercholesterolaemia, the role of LDL/HDL ratio, priority of smoking vs. pharmacological treatment, the desired blood pressure value for diabetics blood pressure and the cut-off value for ten-year risk for primary prevention. For two of the patient cases (case GM with diabetes mellitus, and case AR with a history of angina pectoris), the guidelines allow a simple decision rule to be applied. Of the 32 instances of reference to a rule, 24 were in connection with these two patient cases.

### Risk estimation

For the four primary prevention cases, IS, TW, SH and PU, a number of statements referring to numerical risk estimate (guidelines say 20% within the next ten years) could have been expected. Only two participants referred to numerical risk estimates.

## Discussion

We discuss first how the doctors evaluated the available information in relation to the decision to be made (i.e., in terms of directionality). When each case was analyzed separately, there was some evidence of different patterns of information use shown by prescribers and non-prescribers. The non-prescribers seemed to evaluate central risk factors with a positive directionality less often than prescribers, and they also appeared to identify at least one information category that was given a negative directionality. This is compatible with theories that describe decision-making as search for arguments or reasons for one or the other decision alternative [16,17].

Paradoxically, the information categories that some doctors used as arguments *against *treatment were used as arguments *for *treatment by other doctors. We interpret this finding as showing that prescribing and not prescribing doctors evaluate given information from different perspectives, i.e., from different viewing angles that will put different aspects of the given information in the foreground and background, respectively [18]. If we take smoking or overweight as examples, they could be seen as risk indicators for CHD (which is what is naturally seen from a drug treatment perspective) or as possibilities for life-style change, which in turn will reduce the patient's future coronary risk (which is what is naturally seen from a life-style change perspective). It may be noted that the doctors did in general not consider both ways of evaluating the information to assess their relative weight for and against a decision to prescribe. Instead, only the aspects supporting this decision or an alternative decision were focused, which is in line with the assumption that the doctors viewed the information from a certain perspective that favoured the decision to be made. For example, we can compare the protocol by participant 6 (decision Yes) regarding Case AR: "He has angina and he has overweight so I will treat him" with the protocol from participant 3 (decision No) regarding the same case: "I would like him to reduce his weight first".

The use of life style factors as arguments for prescription decisions was further illustrated in a separate analysis based on the same verbal protocols that also include a task where the doctors were asked to describe freely how they usually reason when they meet patients with high cholesterol values [19]. The protocols were coded for knowledge of guidelines content and for arguments for the decision to prescribe or not. In several instances the doctor seemed to be fully aware of the contents of the guidelines but still decided to refrain from a strict application of it. The arguments for the decisions in these cases often concerned life style factors like smoking or overweight – either as risk increasing factors or as alternative strategies for intervention.

Disagreement was also shown for the age variable. Age is generally considered as positively and monotonically related to risk for future cardiovascular events. At the same time, the guidelines make the reservation that the benefit of giving drugs to very old people is unclear. As far as young patients are concerned, the perspective of ten-year risk appears to be too narrow. The recommended procedure is to increase or project the age to 60 years in order to estimate the risk [4,5]. For doctors with limited experience in using the risk charts, this might be confusing.

Cholesterol was another variable that was ambiguous which could be explained in part by the selection of patient cases. Four of the six cases had cholesterol values in the range of 5.0–6.5 mmol/L, which is often labeled as a mild elevation. This might have formed the basis for negative evaluations, i.e. when a value was close to normal a decision to refrain from drug prescription could have been favored. A few participants also commented that the cholesterol values were lower than they had expected, or lower than those of their own patients.

Ambiguity in the decision situation due to seeing the situation in terms of different treatment perspectives (relevant for life style factors) or different ideas about the optimal cut-off points (relevant for age and laboratory values) could possibly be reduced by clearer guidelines, which in turn accentuate the need for more research on a number of issues. These issues include the role of life-style factors for coronary heart disease, as well as how patients should be motivated to change their life style, and cost-benefit outcomes of using drug treatment of patients in different age groups and with different cholesterol values.

We will now consider the second set of research questions addressed in the present study, viz., the extent to which the participants used certain rules as a basis for their decisions. Based on the verbal protocols, the frequency of statements classified as a rule was rather low, on average 1.6 per participant, and most of the rules concerned secondary prevention. Part of the explanation for the low number of rules might be that the participants were uncertain about the contents of the guidelines and were therefore less willing to talk about them. However, from our separate coding for knowledge of guidelines content referred to above [19], the conclusion was that the doctors were in general well aware of the distinction between primary and secondary prevention.

The low frequency of statements containing a reference to the risk concept could be explained in the same way, since the participants had no immediate access to an aid for calculating risk and were possibly unsure about the general content of such an aid (e.g. the numerical value for ten-year risk that would put the patient into a high-risk category and justify drug treatment). Another reason for the low number of rules might be that the instructions did not encourage the participants to explain their decisions, but simply to state aloud their thoughts about the presented information, which is generally considered as the best method to ensure that the verbal protocols reflect the cognitive processes of interest [13]. A third possible influence on the use of rules may be that cases that could be handled in a straight-forward way by applying rules from the guidelines were at he same time characterized by having cholesterol values that were only marginally increased above normal, which might have introduced conflict in the decision situation.

From the view of evidence-based decisions and quality of care, we can say that many of the cases were difficult and that a considerable spread in the decisions was to be expected. In fact, most of the participants found it difficult to decide about several of the cases, which was evident from interviews after the sessions. On the other hand, the only case (SH) with a mild risk (5–10%) was correctly judged by every participant as not being a candidate for drug treatment. Case AR with angina pectoris represents a decision situation where the guidelines could justify pharmacological treatment in a straightforward manner, and 17 out of 20 chose to prescribe. The presence of diabetes in Case GM could similarly justify drug treatment, but this was the choice for only half the participants. The reason could be that the recommendations concerning diabetes as a risk factor in parity with coronary heart disease is rather new. The Swedish guidelines were published in 1999 and the study was conducted in 2000.

One limitation of the present analyses is that most of the conclusions are based on pooled data from groups of participants, while the principal interest is in strategies at the individual level. For example, the opposing evaluations of the same patient data could only be demonstrated between doctors due to the low number of patient cases. After completion of the six cases, the participants were asked to relate in their own words how they usually reason regarding pharmacological treatment when confronted with patients with high cholesterol values. In a forthcoming paper these narratives will be analyzed at the individual level as "scripts" for dealing with cholesterol treatment. We will then have a better understanding of how knowledge and guidelines in this area of medicine are represented in memory, and how these cognitive structures are related to actual decisions and to the individual doctor's think-aloud protocols from processing the cases.

## Conclusions

In this study we have used a new method to analyse a medical treatment decision. Verbal protocols were coded with respect to how different patient variables seemed to favor or not to favor the decision to prescribe a drug or not. The method promised to be fruitful for understanding why doctors reach different decisions in response to the same patient descriptions and why guidelines are not followed.

## Competing interests

The author(s) declare that they have no competing interests.

## Authors' contributions

All authors participated in the design of the study. LB carried out the data collection. LB and YS performed the coding of the protocols. LB performed the rest of the data analyses and drafted the manuscript. All authors participated in the discussion of the draft. All authors read and approved the final manuscript.

## Pre-publication history

The pre-publication history for this paper can be accessed here:



## Figures and Tables

**Table 1:** Example of a case (IS)

Screen	Information
1	The patient is a 67-year-old woman whose recent cholesterol value was 7.3 mmol/L. She has had the diagnosis hypercholesterolaemia for two years. She has been given advice concerning diet but she has not been prescribed a cholesterol-lowering drug. Her cholesterol value has decreased from an initial value of 7.8 mmol/L
2	The patient has been on medication for hypertension for 10 years (Seloken ZOC* 50 mg and Plendil** 5 mg). She is now on a visit to check her blood pressure and hypercholesterolaemia.
3	The patient has no other known diseases apart from osteoarthritis of her knees. Her mother suffered from hypertension and reached the age of 84 years.
4	The patient is a non-smoker. She very seldom drinks alcohol. She does not exercise on a regular basis but she is fond of taking walks.
5	Physical examination: Good general condition. A few kilograms overweight. Blood pressure 145/75. Heart and lung auscultation normal.
6	Laboratory values: Total cholesterol 7.3 mmol/L. LDL 5.4 mmol/L. HDL 1.2 mmol/L. Triglycerides 1.6 mmol/L. TSH, creatinine and liver-function tests were normal.
7	Would you prescribe a cholesterol-lowering drug for this patient? Yes No

**Table 2:** Characteristics of the cases and the number of doctors who decided to prescribe a drug

Information category	Case					
	
	IS	GM	TW	SH	AR	PU
Age	67	53	67	51	56	41
Sex	Female	Female	Male	Female	Male	Female
Heredity	Slight	Slight	No	Slight	No	Strong
Hypertension	Yes	No	Yes	No	No	No
Diabetes	No	Yes	No	No	No	No
CHD	No	No	No	No	Yes	No
Smoking	No	No	Yes	No	No	No
Overweight	Slight	No	No	No	Yes	No
HDL	1.2	1.1	1.0	1.0	0.9	1.2
LDL	5.4	4.1	3.6	4.3	4.3	5.3
Cholesterol	7.3	5.9	6.0	6.5	5.9	7.2
Decision according to guidelines	No	Yes	Yes	No	Yes	No?*
Percentage of doctors who decided to prescribe	60	50	35	0	85	70

**Table 3:** The frequency of statements with positive or negative directionality. Summary of test statistics The table summarizes the results of ANOVA with the frequency of evaluative statements as dependent variable and Decision, Direction and Information as independent variables. F-values are given with the results of significance tests within parentheses.

	Case					
Statistical effects	IS	GM	TW	SH	AR	PU
Decision	.73 (ns)	1.86 (ns)	.87 (ns)	*	.01 (ns)	.01 (ns)
Direction	10.54 (<.01)	3.98 (.06)	6.09 (<.05)	7.39 (<.01)	.84 (ns)	8.31 (<.01)
Information	7.03 (<.01)	16.80 (<.01)	11.17 (<.01)	1.99 (.09)	5.87 (<.01)	11.15 (<.01)
Decision × Direction	2.55 (ns)	.69 (ns)	7.89 (<.05)	*	12.65 (<.01)	2.42 (ns)
Direction × Information	6.40 (<.01)	5.78 (<.01)	4.57 (.01)	2.18 (.07)	6.28 (<.01)	12.90 (<.01)
Decision × Information	2.06 (<.09)	.61 (ns)	.74 (ns)	*	4.38 (<.01)	1.15 (ns)
Decision × Direction × Information	1.54 (ns)	.85 (ns)	2.18 (ns)	*	3.05 (<.05)	1.09 (ns)
